# Push-out bond strength of zinc oxide eugenol sealer, bioceramic sealer and bioactive glass sealer to dentin among heat generated thermoplasticized gutta-percha technique

**DOI:** 10.6026/973206300223624

**Published:** 2026-06-30

**Authors:** Ayushi Upadhayay, Pankaj Mishra, Santosh Kumar Singh, Shubhashree Rajput, Ashee Jain, Kehkashan Sheikh

**Affiliations:** 1 Department of Conservative Dentistry and Endodontics, People's College of Dental Sciences and Research Centre, People's University, Bhopal, Madhya Pradesh, India

**Keywords:** Push-out bond strength, thermoplasticized gutta-percha, bioceramic sealer, bioactive glass sealer, zinc oxide eugenol sealer

## Abstract

Heat produced during thermoplasticized obturation negatively impacts root canal sealers, compromising their adhesion to dentin and ultimately affecting the integrity of the seal. Therefore, it is of interest to assess the push-out bond strength of zinc oxide eugenol sealer, bioceramic sealer and bioactive glass sealer following thermoplasticized gutta-percha obturation. Sixty single-rooted premolars were randomly divided into three groups and samples from the apical, middle and cervical thirds were evaluated using universal testing equipment. Hence, the bioactive glass sealer showed the greatest push-out bond strength, followed by the bioceramic sealer, whereas the zinc oxide eugenol sealer revealed the lowest values. In all three groups, the apical third consistently exhibited higher bond strength than the middle and cervical thirds. Thus, data shows the performance of traditional and contemporary sealers during heat-assisted obturation and support the potential benefits of bioactive components in enhancing sealer-dentin adhesion.

## Background:

Effective endodontic therapy relies on comprehensive cleaning, shape, disinfection and three-dimensional obturation of the root canal system. Obturation seeks to encapsulate remaining bacteria, avert reinfection and establish a robust seal at the apical, lateral and coronal regions of the prepared canal. Root canal sealers are essential to this goal as they fill the space between gutta-percha and dentinal walls, enhance adaptability and bolster the integrity of the obturation mass [1, 2 3]. The quality of the sealer-dentin interface directly influences leakage resistance, dislodgement resistance and long-term clinical stability [2, 4]. Push-out bond strength is commonly employed to assess the adherence of obturation materials to radicular dentin, as it offers a reliable measure of resistance to dislodgement under standardized settings [3, 4]. Despite its limitations, the push-out method is widely regarded as a reliable laboratory test for evaluating sealers and obturation techniques, particularly when analyzing sections from distinct root levels independently [3]. Variations in dentin structure, tubule density, canal design and sealer penetration across regions may affect bond values, prompting comparisons of the apical, middle and cervical thirds in bond strength research [3]. Traditional zinc oxide eugenol sealers have been utilized for decades due to their handling characteristics and antibacterial qualities. Nonetheless, their bond to dentin is typically regarded as weaker to that of contemporary calcium silicate-based systems [2, 4]. Bioceramic sealers have garnered significant interest due to their hydrophilic properties, dimensional stability, bioactivity, alkaline pH and capacity to generate hydroxyapatite-like interfacial products that may enhance dentin adaption [5, 6, 7, 8]. These materials are progressively utilized alongside single-cone or warm obturation procedures, while their thermal sensitivity continues to be a topic of ongoing research [6, 7 8]. Bioactive glass-based sealers are a promising category of endodontic biomaterials. These materials are engineered to facilitate mineral deposition, chemical interaction with dentin and advantageous biological behavior. Experimental research indicates that bioactive glass-based sealers can infiltrate dentinal tubules, produce calcified surfaces and exhibit promising mechanical and sealing characteristics [9, 10, 11]. Due to its design to both fill and engage with the adjacent dentin, the push-out bond strength of these sealers is a notably significant characteristic [10, 11]. The obturation procedure may also affect sealer efficacy. Warm vertical compaction and other thermoplasticized gutta-percha techniques enhance the adaptability of gutta- percha; nevertheless, the heat produced during these processes may affect the setting reaction, viscosity, film thickness, or interfacial properties of certain sealers [1, 6, 7 , 8]. DeLong *et al.* indicated that continuous-wave obturation may diminish the bond strength of specific calcium silicate sealers; whereas Hoppe *et al.* demonstrated that thermo compaction negatively impacted the long-term bond strength of methacrylate-based sealers [6, 7]. Conversely, studies also noted that a premixed bioceramic sealer preserved advantageous bond strength during warm vertical compaction and recent studies indicate that heat may not substantially compromise bioceramic sealer adhesion under certain conditions [8 , 12]. Simultaneously, comprehensive reviews and rigorous studies suggest that material composition and obturation technique should be evaluated collectively rather than independently [5, 9]. Therefore, it is of interest to analyze and compare the push-out bond strength of three sealers to rootdentin and to examine their performance in the apical, middle and cervical thirds of the root.

## Methodology:

The study was conducted as an *in vitro* experimental examination. Sixty extracted human single-rooted premolars of comparable size were chosen for the investigation. Teeth removed for periodontal or orthodontic purposes were included. All chosen teeth exhibited no cavities, restorations, abrasion, cracks, fracture lines, prior endodontic treatment, or aberrant root morphology. Soft tissue tags and calculus were mechanically excised and the specimens were preserved in normal saline until utilized. The sixty specimens were randomly allocated into three equal groups of twenty teeth each, based on the sealer employed. Specimens in Group 1 were sealed with zinc oxide eugenol, Group 2 specimens were sealed with bioceramic and Group 3 specimens were sealed with bioactive glass. All teeth underwent the preparation of standardized access cavities utilizing an endodontic access bur. Canal patency was verified using a size 10 K-file and the working length was established radiographically. Biomechanical preparation was conducted via rotary instruments up to F3, ensuring uniformity in apical preparation and taper across all specimens. Irrigation with 3% sodium hypochlorite was conducted after each file during instrumentation. Prior to obturation, the canals were irrigated with 17% EDTA, followed by 3% sodium hypochlorite, both utilizing sonic activation and concluded with a final rinse of sterile distilled water. The canals were subsequently dried using sterile paper points. All specimens were sealed utilizing a thermoplasticized gutta-percha approach to ensure uniformity in the thermal effects produced during the obturation process across all groups. The designated sealer for each group was utilized in accordance with the grouping protocol. Subsequent to obturation, each root was transected perpendicular to its longitudinal axis to procure slices indicative of the apical, middle and cervical thirds. Uniform portions were fabricated for push-out testing utilizing a diamond disc. Each specimen underwent push-out bond strength assessment utilizing a universal testing machine, with the dislodging force quantified in megapascals. Failure modes were evaluated and classified as adhesive, cohesive, or mixed.

## Results:

The study comprised 60 removed single-rooted premolars. The samples were evenly allocated into three groups according to the sealer employed: Group 1-zinc oxide eugenol sealer, Group 2-bioceramic sealer and Group 3-bioactive glass sealer. Each root was next divided into apical, middle and cervical thirds and the push-out bond strength was assessed for all portions. At all three root levels, a statistically significant difference was seen among the three groups. In the apical, middle and cervical thirds, the highest mean push-out bond strength was seen in Group 3, followed by Group 2, while Group 1 showed the lowest values (Table 1). This shows that bioactive glass sealer had the best bond strength, followed by bioceramic sealer and then zinc oxide eugenol sealer. In each group, the apical third exhibited the strongest push-out bond strength, succeeded by the middle third and cervical third, with this difference being statistically significant. Upon comparison of the entire group means, Group 3 exhibited the strongest bond strength, succeeded by Group 2 and Group 1 (Table 2) upon aggregating all groups by root level, the apical third exhibited the highest mean value, succeeded by the middle and cervical thirds. The current investigation demonstrated that both the sealer type and the root level significantly influenced push-out bond strength. At each root level, bioactive glass sealer had the highest bond strength; bioceramic sealer demonstrated intermediate values and zinc oxide eugenol sealer presented the lowest values. Consistently, within each sealer category, the apical third exhibited the greatest bond strength, succeeded by the middle third and subsequently the cervical third. The results indicated that the bioactive glass sealer exhibited the highest performance, succeeded by the bioceramic sealer, whilst the zinc oxide eugenol sealer demonstrated the lowest bond strength.

## Discussion:

The current investigation showed that push-out bond strength varied significantly by sealer type and root level. The bioactive glass sealer exhibited the highest overall bond strength, followed by the bioceramic sealer, whereas the zinc oxide eugenol sealer demonstrated the lowest values. The apical third consistently exhibited greater dislodgement resistance than the middle and cervical thirds. This pattern substantiates the notion that both composition-related interfacial chemistry and root-level architecture affect sealer adhesion to dentin. The exceptional efficacy of the bioactive glass sealer in this investigation can be attributed to its bioactivity and mineral-interactive properties. Bioactive glass compositions are recognised for their ability to facilitate ion release, enhance surface mineralisation and stimulate the deposition of apatite-like structures at the dentin interface, potentially improving interlocking and interfacial continuity. Experimental evidence indicates that bioactive glass-based sealers can infiltrate dentinal tubules, produce mineralised interfacial layers and exhibit favourable adhesion qualities [13, 14]. These characteristics likely contributed to the elevated push-out values noted in the current investigation. The bioceramic sealer demonstrated commendable binding strength; however, it was inferior to that of the bioactive glass sealer in this dataset. Calcium silicate -based materials engage with moisture in dentin, liberate calcium ions and generate interfacial reaction products that may improve adaptation. The binding strength of various bioceramic sealers is inconsistent and influenced by formulation, setting characteristics, obturation methods and testing parameters. Merfea *et al.* identified notable disparities between hydraulic calcium silicate sealers and epoxy resin sealers, emphasising that not all calcium silicate-based products exhibit uniform behaviour during push-out tests [1]. Karobari *et al.* similarly observed variability among commercially available sealers, with certain materials exhibiting higher bond strength and more favourable adhesive patterns than others [15]. The current data support the notion that "bioceramic" constitutes a broad category rather than a singular mechanical profile. The comparatively low results observed using zinc oxide eugenol sealer in the present study are likewise justifiable. Traditional zinc oxide eugenol sealers predominantly depend on mechanical adaptation rather than active mineral contact with dentin. Their adhesion is typically inferior to that of contemporary calcium silicate-based or bioactive systems, particularly when accounting for heat, shrinkage and contact imperfections. Recent *in vitro* evaluations of zinc oxide-based materials have demonstrated inferior dislodgement resistance relative to newer formulations [16 , 17]. This finding coincides with the ongoing transition towards bioactive and hydraulic materials to enhance sealer-dentin interaction. A significant observation was the gradual decrease in bond strength from the apical to the middle to the cervical thirds. This regional pattern has been documented in earlier push-out studies and may correlate with variations in dentinal tubule quantity, sclerosis, canal taper, compaction pressure and root section geometry [18]. Similarly, Ozyurek and Turker demonstrated that the behaviour of sealers within the canal is affected by both the characteristics of the material and the canal environment over time when investigating fracture-related outcomes [19]. The elevated apical values in this investigation may indicate enhanced adaptation in narrower canal spaces and improved confinement during thermoplasticized obturation. The failure mode analysis in this investigation corroborated the bond strength results. Adhesive failure was predominant in the zinc oxide eugenol group, while mixed and cohesive failures were more prevalent in the bioceramic and bioactive glass groups. Enhanced interfacial bonding frequently correlates with a decrease in purely adhesive failure and a transition towards cohesive or mixed fracture modes. This association has been documented in experimental literature investigating push-out behaviour and adhesive patterns of root canal sealers [15, 16]. Consequently, the current failure mode pattern reinforced the conclusion that the newer sealers produced more stable sealer-dentin interactions than the conventional material. The current study must be understood within the constraints of an *in vitro* design. Extracted teeth cannot replicate the biological diversity, moisture conditions, periodontal ligament simulation and prolonged temperature and functional stresses of the oral environment. Variations in section thickness, plunger dimensions, storage conditions and material handling may also affect absolute bond strength results. Nonetheless, uniform specimen preparation and consistent testing among all groups enhance the internal validity of the comparisons. Current studies indicate that a bioactive glass sealer may provide a mechanical benefit following thermoplasticized obturation, with the apical third being the most advantageous area for bond retention.

## Conclusion:

We show that the both sealer type and root level greatly affected push-out bond strength to dentin the bioactive glass sealer exhibited the highest bond strength, followed by the bioceramic sealer, whereas the zinc oxide eugenol sealer showed the lowest. In all groups, the apical third had the highest bond strength, followed by the middle and cervical thirds. Data shows that contemporary bioactive sealers may offer superior adherence to root dentin following thermoplasticized obturation and may be advantageous when higher interfacial stability is required.

## Figures and Tables

**Table 1:** Intergroup comparison of push-out bond strength at different root levels

**Root level**	**Group1(Zinc oxide eugenol) Mean ±SD**	**Group2(Bioceramic)Mean ± SD**	**Group3(Bioactive glass)Mean ±SD**	**F value**	**p value**
Apical third	3.8400 ± 0.5200	5.4100 ± 0.6300	6.2800 ± 0.7100	78.64	<0.001*
Middle third	3.3100 ± 0.4700	4.8800 ± 0.5500	5.7600 ± 0.6600	92.15	<0.001*
Cervical third	2.7900 ± 0.4300	4.2600 ± 0.5100	5.1200 ± 0.5800	105.42	<0.001*
*Significant

**Table 2:** Intragroup and overall comparison of push-out bond strength

**Group/Root level**	**Apical Mean ±SD**	**Middle Mean ±SD**	**Cervical Mean ± SD**	**Overall Mean ± SD**	**F value**	**p value**
Group 1-Zinc oxide eugenol	3.8400 ± 0.5200	3.3100 ± 0.4700	2.7900 ± 0.4300	3.3133 ± 0.6100	24.83	<0.001*
Group 2-Bioceramic	5.4100 ± 0.6300	4.8800 ± 0.5500	4.2600 ± 0.5100	4.8500 ± 0.7400	21.76	<0.001*
Group 3-Bioactive glass	6.2800 ± 0.7100	5.7600 ± 0.6600	5.1200 ± 0.5800	5.7200 ± 0.8200	19.98	<0.001*
Overall by root level	5.1767 ±1.1200	4.6500 ± 1.0400	4.0567 ± 0.9700	-	26.49	<0.001*
*Significant
